# Adopting Ambulatory Breast Cancer Surgery as the Standard of Care in an Asian Population

**DOI:** 10.1155/2014/672743

**Published:** 2014-08-12

**Authors:** Yvonne Ying Ru Ng, Patrick Mun Yew Chan, Juliana Jia Chuan Chen, Melanie Dee Wern Seah, Christine Teo, Ern Yu Tan

**Affiliations:** Department of General Surgery, Tan Tock Seng Hospital, 11 Jalan Tan Tock Seng, Singapore 308433

## Abstract

*Introduction*. Ambulatory surgery is not commonly practiced in Asia. A 23-hour ambulatory (AS23) service was implemented at our institute in March 2004 to allow more surgeries to be performed as ambulatory procedures. In this study, we reviewed the impact of the AS23 service on breast cancer surgeries and reviewed surgical outcomes, including postoperative complications, length of stay, and 30-day readmission. *Methods*. Retrospective review was performed of 1742 patients who underwent definitive breast cancer surgery from 1 March 2004 to 31 December 2010. *Results*. By 2010, more than 70% of surgeries were being performed as ambulatory procedures. Younger women (*P* < 0.01), those undergoing wide local excision (*P* < 0.01) and those with ductal carcinoma-in situ or early stage breast cancer (*P* < 0.01), were more likely to undergo ambulatory surgery. Six percent of patients initially scheduled for ambulatory surgery were eventually managed as inpatients; a third of these were because of perioperative complications. Wound complications, 30-day readmission and reoperation rates were not more frequent with ambulatory surgery. *Conclusion*. Ambulatory breast cancer surgery is now the standard of care at our institute. An integrated workflow facilitating proper patient selection and structured postoperativee outpatient care have ensured minimal complications and high patient acceptance.

## 1. Introduction

Ambulatory surgery was initially limited to procedures performed under local or regional anaesthesia, which required minimal postoperative monitoring. Anesthesia techniques and perioperative management have evolved ever since such that low risk surgeries performed under general anaesthesia can now also be performed in the ambulatory setting. Patients undergoing breast cancer surgery seldom develop serious complications and most return to their preoperative function soon after the surgery, making them ideal candidates for ambulatory surgery. In spite of this, many patients have been managed in the past as inpatients due to concerns about drain care and the lack of structured outpatient follow-up care. This has gradually changed over the years. After sentinel lymph node biopsy (SLNB) was adopted as the standard of care, full axillary lymph nodal dissection (ALND), and consequently the use of surgical drains, became less common. This, together with the establishment of specialised breast units in many centres to provide continuity of care after hospital discharge, has led to a greater push towards ambulatory breast cancer surgery.

Early discharge has been shown to contribute to greater healthcare efficiency without compromising the quality of care. Studies have consistently affirmed the safety and benefits of ambulatory surgery, even in patients discharged with surgical drains* in situ* [1–3]. Despite this, ambulatory surgery is not as readily accepted in Asia as compared to Western countries, where there is greater emphasis on patient empowerment [4, 5]. Older women, in particular, are reluctant to be discharged home early as they perceive cancer surgery to be major surgery and believe that specialised care in a hospital setting during the postoperative period will prevent complications and even future disease relapse.

In March 2004, our institute introduced a 23-hour ambulatory surgery service (AS23). The AS23 unit functions as an independent facility from the inpatient wards, with its own bed capacity and staff complement. Strict admission criteria ensure that only patients undergoing low risk surgeries under general anaesthesia and who require only basic postoperative monitoring and care are admitted. Patients are typically admitted to the unit after surgery and are monitored until they are discharged home, either later on the same day or the following morning. Patients who develop perioperative complications requiring more intensive monitoring or who cannot be discharged by the following morning are transferred to the inpatient wards. Women undergoing breast cancer surgery were among the first to be included in this service. Prior to this, all women were admitted to the inpatient wards after breast cancer surgery. In this study, we reviewed the outcomes of women who underwent breast cancer surgery at our institute over a 7-year period, starting from the implementation of the AS23 service. In order to determine the safety and feasibility of ambulatory breast cancer surgery, we evaluated the frequency of postoperative complications, the frequency of unplanned prolonged hospital stays, and the readmission rate within 30 days of surgery. Patient and disease factors favouring ambulatory surgery were also identified.

## 2. Materials and Methods

A retrospective review was performed of 1742 women who underwent definitive breast cancer surgery at our institute from 1 March 2004 to 31 December 2010. This study has ethical committee approval (2011/00410). A total of 1822 breast cancer surgeries were performed during this period. These included 18 bilateral procedures (bilateral mastectomy or wide local excision (WLE), with or without SLNB or ALND) and 62 repeat surgeries for mastectomy or ALND. Those who underwent immediate breast reconstruction were also included. Surgeries were performed either as a day surgery (DS) procedure (with patients being discharged on the same day of surgery), an AS23 procedure (patients were discharged the following morning), or as an inpatient procedure (patients were discharged more than 24 hours after surgery). Both DS and AS23 procedures were considered ambulatory surgery. A single drain would be inserted under the skin flaps following a mastectomy, and another into the axilla following ALND. Surgical drains were not inserted if a WLE or a SLNB was performed.

Following surgery, all patients were transferred and monitored in the Postanesthesia Care Unit (PACU). After the discharge criteria were satisfied, patients were then transferred to the AS23 unit or inpatient wards. Diet and long-term medications, with the exception of anticoagulants and antiplatelet agents, were resumed once the patients were fully awake. Oral analgesia was prescribed for pain relief, while antiemetics were given on a* pro re nata* basis. Prior to discharge, patients were reviewed by the surgical team, and specialist breast care nurses would reinforce instructions on wound, drain care, and arm physiotherapy. The nurses would also schedule another review in the outpatient clinic 3 to 4 days later.

The decision for surgery was made following discussions between the patient and surgeon. Patients were scheduled for ambulatory surgery unless they had existing medical conditions that necessitated more intensive postoperative monitoring or if they were undergoing immediate breast reconstruction (breast reconstruction with autologous myocutaneous flaps is standard at our institute). Those with poor family or social support and who were residents of nursing homes or mental institutes were also managed as inpatients. Specialist breast care nurses would then engage patients and their families in preoperative counselling sessions, where the surgical process, postoperative recovery, and concerns regarding early discharge were discussed. Thereafter, patients were evaluated by the anaesthesia team to assess the suitability for ambulatory surgery and to optimise the control of any existing comorbidities.

Data collected included age, ethnicity, preexisting medical conditions, tumor characteristics, surgical procedure, and postoperative outcomes including complications, length of hospital stay, and readmissions within 30 days of discharge. Comparison was made between those who had undergone ambulatory versus inpatient surgery. Correlation analyses were performed using the chi-square test or Fisher's exact test where appropriate; the Mann Whitney *U* test was used to compare median age and median length of stay. Statistical analysis was performed with GraphPad Prism version 5.0 (GraphPad software Inc., San Diego CA). A 2-tailed *P* value test was used in all analyses and a *P* value of less than 0.05 was considered statistically significant.

## 3. Results

A total of 1742 women underwent definitive breast cancer surgery at our institute in the 7-year period. Median patient age was 54 years (ranging from 20 to 94 years) and ethnic distribution reflected that of local population demographics (Table 1). Eighty-three percent of surgeries were performed for invasive carcinoma, and 73.1% were classified as Stage I or Stage II disease. Of the 1822 surgical procedures, 1277 (70.1%) were done in the ambulatory setting, either as day surgery procedures or AS23 procedures (Table 2). Those who underwent WLE were 6 times more likely to undergo surgery in the ambulatory setting compared to those who underwent mastectomy (*P* < 0.01; OR 5.89; 95% CI 4.56–7.61) (Table 1). Ambulatory surgery was also more common in younger women and those with less advanced disease (*P* < 0.01 and *P* < 0.01; OR 2.30; 95% CI 1.81–2.92). The apparent association with disease stage resulted from a significantly greater proportion of women with DCIS and Stages I and II cancers undergoing WLE (684 of 1299 such women underwent WLE), compared to those with Stages III and IV cancers (where 54 of 368 such women underwent WLE). There was no association with ethnicity. Over the median follow-up period of 53 months (ranging from 2 to 102 months), disease recurrence (locoregional and/or distant) developed in 199 of 1742 patients (11.4%). Although recurrent disease appeared more common among those who had been managed as inpatients, this was no longer significant after adjusting for disease stage (*P* > 0.05).

**Figure 1:**
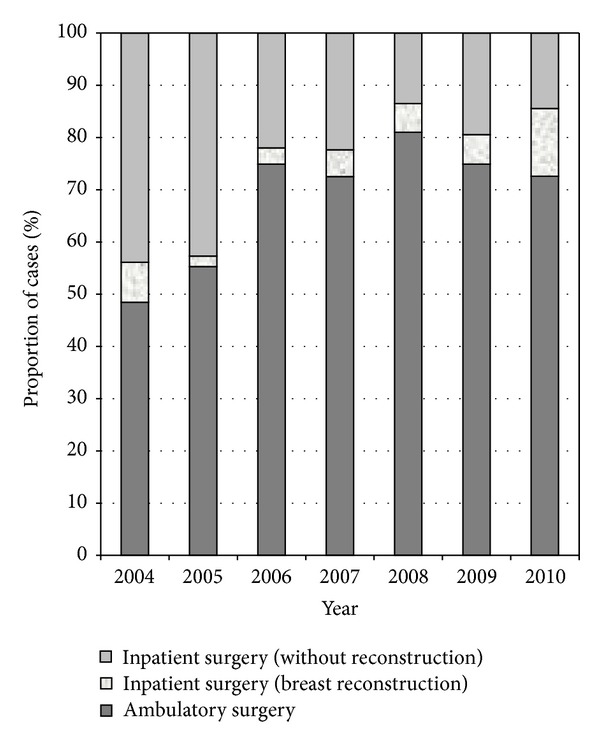
Proportion of surgeries being performed as ambulatory and inpatient procedures from 1 March 2004 to 31 December 2010.

There has been an increasing trend towards ambulatory surgery over the 7 years (*P* < 0.01) (Figure 1). In the first year of implementation, ambulatory surgery constituted 49% of all breast cancer surgeries performed in our institute. By 2006, more than 70% of surgeries were being performed in the ambulatory setting. The proportion of surgeries done as ambulatory procedures did not increase much further after the third year. Correspondingly, we also observed that the numbers of inpatient surgeries involving immediate breast reconstruction had also increased (*P* < 0.01); almost half the inpatient surgeries performed in 2010 involved breast reconstruction.

Seventy-five patients (6.2%) who were initially scheduled for ambulatory surgery were managed as inpatients instead. About a third (28 of 75, 37.3%) of these patients had requested to stay longer because family members were not confident of caring for them. Sixteen patients (21.6%) stayed more than 24 hours because of persistent giddiness, nausea, and postural hypotension, which all resolved with conservative treatment within the next 2 days. Details are included in Table 3. All patients had received the standard drugs perioperatively and there were no perioperative events that could have precipitated the postanaesthesia events. These patients tended to be older (median age in this group was 63 years, compared to the median age of 54 years for the entire cohort); 10 patients had preexisting comorbidities which were well optimised and 6 had no known medical history. Twenty-nine patients (38.7%) were admitted to the inpatient wards for more intensive monitoring following unanticipated perioperative events. Seventeen patients developed perioperative events such as cardiac arrhythmias, chest pain, desaturation, or allergic reactions to anaesthetic drugs, and 12 patients developed wound complications such as persistent wound site bleeding or pain and dislodged or blocked drains; four of these 12 patients required an unplanned operation for wound exploration and haemostasis. Details are included in Table 4. Median age of these patients was 61 years (ranging from 42 to 83 years). All patients were haemodynamically stable throughout the surgery, although 1 patient had persistently elevated blood pressure that required intravenous esmolol for control, and 2 patients were noted to have new cardiac arrhythmias. Two patients who developed blue dye allergy had transient hypotension which responded to fluids. One of the 10 patients who developed postoperative wound bleeding was on long-term aspirin for primary prevention, but this had been discontinued 7 days prior to surgery as per standard protocol. The tumour was staged as T1 in 3 patients, as T2 in 7 patients, and as T3 in 2 patients; and none of the tumours were adherent to the underlying pectoralis muscle, which was preserved in all cases. There was no documented episode of sustained hypertension in the recovery period that could have predisposed to bleeding. Wound exploration was performed in the 4 patients based on a suspicion of ongoing active bleeding, in view of associated hypotension and anaemia requiring blood transfusion despite wound compression. In 1 patient, a dislodged haemostatic clip was found to be the cause of the bleeding, while in the other 3 patients who underwent wound exploration, there were no active bleeders but only a slow generalised ooze. Two patients were admitted for evaluation of incidentally detected nonsurgical related problems (incidental renal stones and lower limb cellulitis, resp.). Median length of stay in excess of that originally scheduled was 2 days (ranging from 1 to 22 days). 24 patients were admitted for longer than 2 additional days; 15 were patients who had developed unanticipated postoperative events while the remaining 9 remained in hospital for social reasons.

One patient in the ambulatory surgery group developed a major postoperative complication. This patient had undergone mastectomy and insertion of implant as an ambulatory procedure and developed an acute cerebral infarct in the immediate postoperative period. She had no predisposing factors other than hypertension, which had been well-controlled prior to the surgery. Surgery had also been uneventful. Antiplatelet therapy was started and she was subsequently transferred to a neurology rehabilitation unit where she made a full functional recovery. Sixty-two patients (3.6%) were readmitted within 30 days of surgery, 37 (59.6%) of whom had undergone ambulatory surgery (Table 5). The majority of patients who were readmitted had undergone mastectomy (26 as an ambulatory procedure and 24 as an inpatient); this included 7 patients who had undergone breast reconstruction. Wound complications such as bleeding and infection were the most common reasons for readmission and 15 patients required surgical intervention. Wound complications were not more common among those with ambulatory surgery, and, of note, there were no readmissions for drain-related issues. Ambulatory surgery was not associated with readmission within 30 days of surgery, nor did it increase the risk of reoperation for postoperative complications (*P* > 0.05). Median length of stay during the readmission episode was also similar between the two groups.

## 4. Discussion

Hospital stays after breast cancer surgery were shortened after it became apparent that early discharge, even with the surgical drains* in situ*, was safe and did not compromise recovery [3, 6–11]. This eventually evolved into the concept of ambulatory surgery. While ambulatory surgery has become well accepted in many Western countries, it is less commonly practiced in Asia [2, 3, 6, 7, 10, 12–16]. Early discharge after surgery involves a major change in patient mindset, requiring them to be confident of recovery outside of what is often perceived as a more controlled and specialised environment. More importantly, early discharge is possible only when there is adequate home and social support and a well-organised infrastructure to provide professional and comprehensive postoperative outpatient care.

Our institute was one of the first in Singapore to actively push for breast cancer surgery to be done as an ambulatory procedure. Since the first implementation of the AS23 service in 2004, more than 70% of all breast cancer surgeries are now being performed as ambulatory procedures in our institute. Ambulatory breast cancer surgery is now considered the norm, rather than the exception. Only 6% of those initially scheduled for ambulatory surgery were not discharged as planned; even so, most stayed only for an additional 2 days. Most of our patients had adequate home support, and only 2% of patients opted for inpatient admission because of social reasons. Similar to other published reports, we have observed that patient safety was not compromised by early discharge [1–3]. Readmission within 30 days of surgery was not more common among those who had undergone ambulatory surgery. Wound complications, such as wound haematoma and infection, were the most common reasons for readmission but were not more frequent nor more severe in those who had ambulatory surgery, implying that wound care in the outpatient setting was comparable to that provided in the hospital wards. Even when wound complications developed, most resolved with conservative management and very few patients required further surgery. Of note, none of the patients who had been discharged with drains* in situ* were readmitted for drain-related complications.

Psychological benefit and improved patient outcomes have been said to be among the main advantages of ambulatory surgery [3, 12]. Many Asian women, the elderly in particular, are apprehensive about early discharge because cancer surgery is thought to take such a physical toll on the body that recovery would be slow and difficult. On the other hand, advising that surgery be done as an ambulatory procedure can instead give the impression that the surgery is likely to be straightforward and uncomplicated. This may in turn promote better emotional and psychological adaptation and a faster return to normal activities, explaining why better outcomes are observed.

Yet another major benefit of ambulatory surgery is the significant cost savings resulting from shorter hospital stays [8, 10–13, 17–20]. This has particular relevance in our local context. The initiative for an AS23 service to facilitate ambulatory surgery was largely driven by increasing pressure on hospital beds [21]. Faced with an ageing population, local hospitals are seeing a significant increase in the number of elderly patients being admitted. Elderly patients are more often frail and, with multiple medical problems, they take longer to recover and are particularly vulnerable to deconditioning [22, 23]. Loss of functional independence further slows bed turnover as families are not always able to cope with the additional care needed and these patients then have to remain in hospital until transfer to a step-down care facility. In recent years, it has become increasingly common to have to reschedule elective surgeries because the hospital bed capacity has been exceeded. Rescheduling is psychologically frustrating and inconvenient for the patient and their families and also adds to the workload of the healthcare staff involved. The AS23 service was set up to provide additional resources to allow surgery to proceed as scheduled regardless of inpatient bed availability. Its implementation has led to more streamlined workflows to allow more patients to undergo ambulatory surgery without having to be admitted to the inpatient wards. This has helped to free up inpatient beds for acute admissions. An integrated workflow involving the surgeon, anaesthetist, and the breast care nurse specialist ensures proper patient selection and realistic management of patient expectations. Surgical and anaesthetic reviews ensure patient safety by selecting only the patients who are not expected to be at an increased risk of perioperative complications. While these medical and administrative aspects are essential, we would not have been able to achieve such a high uptake of ambulatory surgery among our patients without the active involvement of our specialist breast care nurses [10]. The breast care nurses are primarily responsible for counselling and postoperative wound and drain care and are instrumental in providing the necessary support for postoperative outpatient care. The rare occurrence of serious complications and the continuity of care provided by the breast care nurses have undoubtedly contributed towards reassuring our patients and their family of the safety and feasibility of ambulatory surgery.

## 5. Conclusion

Our study has shown that ambulatory breast cancer surgery can be successfully implemented in an Asian population. Patient acceptance is high while postoperative complication and readmission rates are low. An integrated workflow involving the surgeons, anaesthetists, and breast care nurses is fundamental to the success of ambulatory surgery. Such a workflow minimises the occurrence of adverse events through proper patient selection and ensures continuity of care upon discharge.

## Figures and Tables

**Table 1:** Correlation analyses comparing between women who had undergone ambulatory surgery and women who had undergone inpatient surgery (*n* = 1742).

Characteristics	Ambulatory surgery (*n* = 1207)	Inpatient surgery (*n* = 535)	*P* value
Median age (years) (range)	53 (20–91)	57 (23–94)	<0.01
Ethnicity			0.29
Chinese	975	414	
Malay	107	58	
Indian	62	33	
Others	63	30	
Tumour type			0.18
DCIS	237	60	
IDC	857	422	
ILC	56	26	
Others	57	27	
Disease stage			<0.01
DCIS	237	60	
I	326	120	
II	391	165	
III	176	140	
IV	25	27	
Surgical procedure			<0.01
WLE with or without SLNB	415	27	
WLE with ALND	235	61	
Mastectomy with or without SLNB	224	80	
Mastectomy with ALND	314	232	
Bilateral procedures^a^	18	17	
Mastectomy with immediate reconstruction	1^b^	118^c^	
Disease recurrence			<0.01
Yes	110	89	
No	1097	446	

WLE: wide local excision; SLNB: sentinel lymph node biopsy; ALND: full axillary lymph node dissection. ^a^Bilateral mastectomy or WLE. ^b^Insertion of implant. ^c^Autologous flap reconstruction or insertion of implant.

**Table 2:** Details of surgical procedures performed as ambulatory surgery (*n* = 1277).

	Day surgery	AS23
WLE with or without SLNB	309	127
WLE with ALND	37	206
Mastectomy with or without SLNB	19	243
Mastectomy with ALND	7	311
Bilateral procedures^a^	0	18

WLE: wide local excision; SLNB: sentinel lymph node biopsy; ALND: axillary lymph node dissection. ^a^Bilateral mastectomy and WLE.

**Table 3:** Details of 16 patients with postanaesthesia events who were admitted for longer than 24 hours.

Patient	Age (years)	Preexisting comorbidities	Surgery	Event	Management	LOS (days)
1	68	Hyperlipidaemia	Mastectomy/AC	Giddiness	Expectant	2

2	47	Hyperlipidaemia	Mastectomy/AC	Giddiness	Expectant	2

3	41	Thalassemia minor	Mastectomy/AC	Giddiness	Expectant	2

4	59	NIL	Mastectomy/AC	Postural hypotension	Fluid challenge	3

5	69	Hypertension, hyperlipidaemia, previous subdural haematoma	Mastectomy/SLNB	Giddiness	Expectant	2

6	81	NIL	Mastectomy/SLNB	Giddiness	Expectant	2

7	48	NIL	Mastectomy/AC	Giddiness	Expectant	3

8	57	NIL	Mastectomy/AC	Nausea and vomiting	Antiemetics	2

9	71	Hypertension, hyperlipidaemia, diabetes mellitus	WLE/AC	Postural hypotension	Expectant	2

10	48	NIL	Mastectomy/SLNB	Giddiness	Expectant	2

11	67	Hypertension, hyperlipidaemia, diabetes mellitus	Mastectomy/SLNB	Giddiness	Expectant	2

12	79	Hypertension, hyperlipidaemia, diabetes mellitus	Mastectomy/SLNB	Postural hypotension	Expectant	2

13	71	Hypertension, hyperlipidaemia, diabetes mellitus	Mastectomy/SLNB	Giddiness	Expectant	2

14	67	NIL	Mastectomy/SLNB	Giddiness	Expectant	2

15	47	Hypertension, hyperlipidaemia, diabetes mellitus	Mastectomy/AC	Nausea and vomiting	Antiemetics	2

16	54	Hypertension, diabetes mellitus	Mastectomy/AC	Giddiness	Expectant	2

AC: axillary clearance; SLNB: sentinel lymph node biopsy; WLE: wide local excision.

**Table 4:** Details of 29 patients who required inpatient admission for management of unanticipated perioperative events.

Patient	Age (years)	Preexisting comorbidities	Surgery	Event	Management	LOS (days)
1	59	Hypertension, hyperlipidaemia, diabetes mellitus	Mastectomy/AC	Wound bleeding	Wound exploration and haemostasis	4

2	62	Hypertension, hyperlipidaemia, asthma, obstructive sleep apnoea (OSA)	WLE/AC	Desaturation due to OSA and drowsiness postoperatively	CPAP	6

3	66	Hypertension, hyperlipidaemia, diabetes mellitus, previous transient ischaemic attack	WLE	Uncontrolled blood pressure intraoperatively	Expectant	4

4	42	Obesity, obstructive sleep apnoea	Mastectomy/SLNB	New onset of atrial fibrillation	Cardiology consult; beta-blockers	4

5	67	Hypertension, hyperlipidaemia, diabetes mellitus	Mastectomy/SLNB	Wound bleeding	Wound exploration and haemostasis	4

6	62	Hyperlipidaemia	Mastectomy/AC	High drain output	Expectant	2

7	43	Hyperthyroidism	WLE/SLNB	Negative pressure pulmonary edema secondary to laryngospasm after extubation	CPAP and diuretics	5

8	58	Hypertension, hyperlipidaemia	Mastectomy/AC	High drain output	Expectant	2

9	57	NIL	Mastectomy/AC	Wound pain	Expectant	2

10	51	NIL	WLE/AC	Atypical chest pain	Expectant	2

11	82	Hypertension, schizophrenia, aortic sclerosis	Mastectomy/AC	Premature ventricular contractions and hypotension intraoperatively	Expectant	4

12	61	NIL	Mastectomy/AC	Low oxygen saturation postoperatively	Expectant	4

13	78	Hypertension, renal stones	Mastectomy/SLNB	Acute urinary retention	Indwelling urinary catheter	5

14	44	NIL	WLE/SLNB	Atypical chest pain	Expectant	1

15	55	NIL	WLE/SLNB	Intraoperative hypotension secondary to blue dye allergy	Expectant	1

16	60	NIL	Mastectomy/AC	Wound bleeding	Wound exploration and haemostasis	2

17	61	History of atypical chest pain	WLE/SLNB	Atypical chest pain	Expectant	2

18	60	Hypertension, hyperlipidaemia, asthma	Mastectomy/SLNB	High drain output	Expectant	4

19	42	Mitral valve prolapse	Mastectomy/SLNB and laparoscopic myomectomy	Wound (abdominal) pain	Expectant	2

20	65	Hypertension, hyperlipidaemia	Mastectomy/SLNB	High drain output	Expectant	4

21	83	Hypertension, hyperlipidaemia	Mastectomy/AC	Wound pain	Expectant	2

22	67	NIL	WLE/SLNB	Intraoperative hypotension secondary to blue dye allergy	Expectant	1

23	49	Asthma	Mastectomy/AC	Wound bleeding	Expectant	1

24	60	NIL	Mastectomy/SLNB	Coffee ground aspirate intraoperatively	Proton pump inhibitors	4

25	81	Hypertension, hyperlipidaemia, diabetes mellitus	Mastectomy/SLNB	Acute urinary retention	Indwelling urinary catheter	7

26	65	Hepatitis B carrier	Mastectomy/AC	High drain output	Expectant	2

27	79	Diabetes mellitus, ischaemic heart disease	Mastectomy/AC	Mild congestive cardiac failure	Diuretics	6

28	62	Hypertension	Mastectomy/SLNB and implant insertion	Cerebrovascular event	Antiplatelet therapy, neurorehabilitation	22

29	43	NIL	Mastectomy/AC	Wound bleeding	Wound exploration and haemostasis	3

AC: axillary clearance; SLNB: sentinel lymph node biopsy; WLE: wide local excision; CPAP: continuous positive airway pressure.

**Table 5:** Details of surgical outcomes in patients who had undergone ambulatory surgery and inpatient surgery (*n* = 1742).

Characteristics	Ambulatory surgery (*n* = 1207)	Inpatient surgery (*n* = 535)	*P* value
Number of readmissions within 30 days	37	25	0.07
Median length of stay following readmission (days)	3 (1–27)	4 (1–17)	0.07
Complications			0.69
Wound hematoma or bleeding	15	7	
Wound infection	13	9	
Wound dehiscence	0	4	
Wound pain	3	0	
Drain complications^a^	0	5	
Others^b^	6	0	
Number of reoperations	10	5	0.77
Wound exploration and haemostasis	8	1	
Wound debridement	2	1	
Secondary suture of wound	0	3	

^a^High drain output, drain dislodgement. ^b^Syncope in 3 patients, lower limb deep venous thrombosis in 1 patient, diarrhoea in 1 patient, and hyperkalemia in 1 patient.
